# Haloperidol–induced catalepsy is ameliorated by deep brain stimulation of the inferior colliculus

**DOI:** 10.1038/s41598-018-19990-y

**Published:** 2018-02-02

**Authors:** K.-Alexander Engelhardt, Philine Marchetta, Rainer K. W. Schwarting, Liana Melo-Thomas

**Affiliations:** 1 Behavioral Neuroscience, Experimental and Biological Psychology, Philipps-University of Marburg, Gutenbergstr. 18, D-35032 Marburg, Germany; 2 Marburg Center for Mind, Brain, and Behavior (MCMBB), Marburg, Hans-Meerwein-Straße 6, 35032 Marburg, Germany; 3 Behavioral Neurosciences Institute (INeC), Av. do Café, 2450, Monte Alegre, Ribeirão Preto, 14050-220 São Paulo, Brazil

## Abstract

Deep brain stimulation (DBS) has evolved as a promising alternative treatment for Parkinson’s disease (PD), but the underlying mechanisms remain poorly understood. Moreover, conventional DBS protocols targeted at basal ganglia sites can turn out completely ineffective for some PD patients, warranting the search for alternative targets. The inferior colliculus (IC) is a midbrain auditory relay station involved in sensorimotor processes. High-frequency 2500 Hz electrical stimulation of the IC elicits escape behaviour and interferes with haloperidol-induced catalepsy in rats, a state reminiscent of Parkinsonian akinesia, but clinical implication is limited since the protocol is aversive. However, typical DBS stimulation frequencies range between 20–180 Hz. We therefore tested the effects of a low-frequency 30 Hz-DBS protocol on haloperidol-induced catalepsy and aversive behaviour in rats. We show that low-frequency 30 Hz-DBS targeted at the IC strongly ameliorates haloperidol-induced catalepsy without any evidence of stimulation-induced escape behaviour. Furthermore, 30 Hz-DBS of the IC produced no place avoidance in a place conditioning paradigm and induced no anxiety-related behaviour on the elevated plus maze, indicating that the protocol has no aversive or anxiogenic side effects. Our findings provide first evidence that the IC can serve as an alternative, non-conventional DBS target.

## Introduction

Deep brain stimulation (DBS) has evolved as a promising treatment strategy for severe cases of Parkinson’s disease (PD), a neurodegenerative movement disorder characterized by tremor, rigidity, and bradykinesia/akinesia^1^. Current theories assume that PD deficits are driven by pathological network activity in the basal ganglia^2^ and DBS is therefore typically targeted at basal ganglia structures, such as the subthalamic nucleus^3^. However, the underlying mechanisms remain poorly understood^1^. Moreover, conventional DBS protocols targeted at basal ganglia sites can turn out completely ineffective for some PD patients^4^, warranting the search for alternative targets.

The inferior colliculus (IC) is an evolutionary conserved midbrain tectum structure with homologous organization across vertebrate species^5^, consisting of a central nucleus surrounded by an external and dorsal cortex^6,7^. In both humans and rodents, the IC serves as an important relay station for ascending and descending auditory information, but is distinguished from other brainstem auditory nuclei by its indirect projections to motor pathways^5^. Indeed, several studies showed that the IC also participates in sensorimotor processes. For instance, prepulse inhibition of acoustic startle, an operational task of sensorimotor gating, is regulated by dopaminergic mechanisms in the IC^8^. Moreover, we showed that microinjections of glutamatergic drugs into the IC modulate haloperidol-induced catalepsy in rats^9,10^, a state reminiscent of Parkinsonian akinesia^11^. Finally, the IC is considered as part of the brain defence system since electrical stimulation of this structure induces aversive behaviour in a graded manner^12–14^. High-frequency 2500 Hz electrical stimulation of the IC at specific current thresholds elicits escape behaviour^12,13^ and we recently showed that such stimulation at these thresholds also induces vigorous escape responses in rats treated with haloperidol, thereby releasing the cataleptic state^15^. Such an effect is reminiscent of a clinical phenomenon known as paradoxical kinesia, i.e. the sudden and transient ability of akinetic PD patients to perform coordinated movements (e.g. flight reactions) in response to an emotionally significant trigger, such as emotional stress^16^.

These findings suggests that the IC might serve as an alternative DBS target, but clinical implication is limited since 2500 Hz stimulation is aversive. Since typical DBS stimulation frequencies range between 20–180 Hz^3,17,18^, we now asked whether low-frequency 30 Hz-DBS of the IC can reduce haloperidol-induced catalepsy without aversive side effects. We chose 30 Hz stimulation because clinical evidence suggests that lower DBS frequencies might be more effective than higher ones^17^. This is particularly the case for structures located outside the basal ganglia, such as the pedunculopontine nucleus, another brainstem structure involved in motor behaviour besides the IC, where optimal stimulation frequencies range between 20–80 Hz^18,19^.

## Results

### Low-frequency deep brain stimulation of the IC ameliorates akinesia-like motor deficits

While high-frequency, i.e. 2500 Hz stimulation of the IC effectively interferes with haloperidol-induced catalepsy, the effect is achieved by inducing a defence reaction, i.e. escape behaviour^15^, and therefore associated with clear aversive side effects. Moreover, the stimulation frequency is far outside the clinically effective range commonly used in the treatment of PD^3,17–19^.

**Figure 1:**
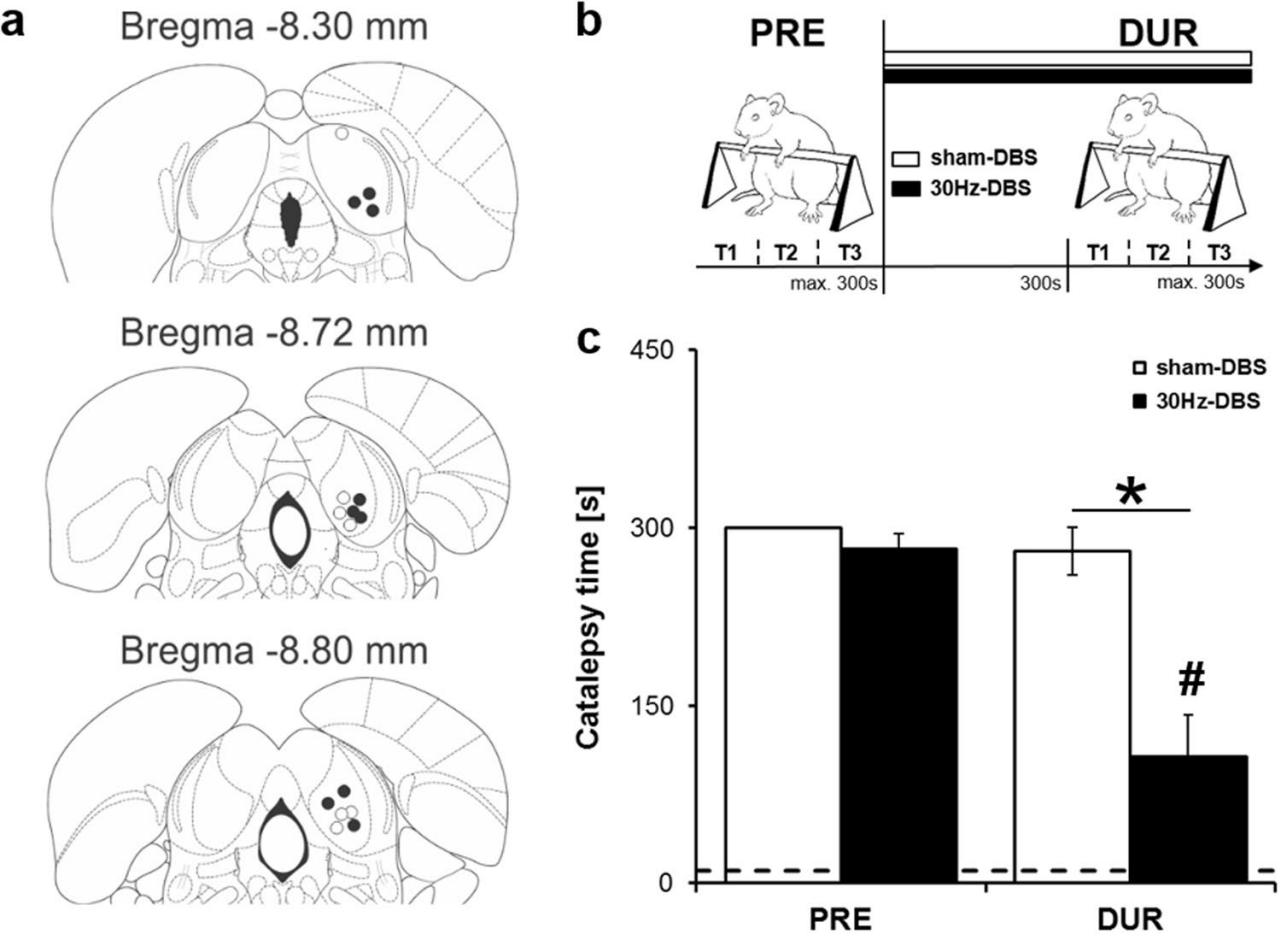
30 Hz deep brain stimulation of the IC ameliorates haloperidol-induced catalepsy. (**a)** Electrode placements in the IC for 30 Hz-DBS (black dots) and sham-DBS (white dots) on coronal sections from the Paxinos and Watson^24^ atlas. **(b)** Illustration of experimental protocol. Haloperidol-induced catalepsy (0.5 mg/kg, i.p.) was first confirmed during a pre-test (PRE) by measuring the step-down latency from a horizontal bar on three consecutive trials, using a 300 s stop criterion. Rats then received sub-chronic 30 Hz-DBS at the IC for 5 min and were submitted to a second catalepsy test under continued stimulation (DUR). The procedure for sham-DBS was identical except that no current was administered. **(c)** Time spent in haloperidol-induced catalepsy before (PRE) or during (DUR) either 30 Hz-DBS or sham-DBS of the IC. The dotted line indicates the control step-down latencies following saline administration (1.0 ml/kg, i.p.) obtained 1 day before testing for haloperidol-induced catalepsy. The saline scores did not differ between 30 Hz-DBS and sham-DBS and were therefore averaged across groups. The time spent in haloperidol-induced catalepsy strongly decreased from PRE to DUR in response to 30 Hz-DBS of the IC, resulting in a clear reduction compared to sham-DBS, where no change was seen. Data are presented as mean ± s.e.m. *n* = 7 for sham-DBS, *n* = 9 for 30 Hz-DBS. ^#^*P* < 0.05 vs. PRE; *P < 0.05 vs. sham-DBS.

We therefore modified our previously established DBS protocol by switching to a low-frequency, i.e. 30 Hz stimulation regime, which is set within the clinically effective frequency range, and tested its effects on haloperidol-induced catalepsy and aversive behaviour in rats compared to sham-DBS control. To this aim, we implanted a stimulation electrode unilaterally into the central nucleus of the IC (Fig. 1a) and first assessed each rat’s escape threshold individually during 2500 Hz stimulation of the IC using our standard protocol^15^. These thresholds, which verify electrode placement within the IC, were determined by successively increasing the current intensity in 20–50 μA steps until reaching the minimal current intensity producing escape behaviour, i.e. intense running or jumping (see Materials and Methods). Escape thresholds could be determined in all rats except two where no escape was seen, possibly due to electrode displacement (Fig. 1a). These rats were therefore assigned to the sham-DBS group. We then switched to 30 Hz stimulation, but kept the individual current intensities of the threshold procedure.

We next treated rats with physiological saline (1 ml/kg, i.p.) or haloperidol (0.5 mg/kg, i.p.) on consecutive days and assessed the effects of our 30 Hz-DBS protocol on the time spent in catalepsy 60 min post-injection by measuring rats’ step down latency from a horizontal bar (Fig. 1b). Rats were first stimulated sub-chronically for 5 min with 30 Hz-DBS of the IC (*n* = 9) administered at individual current thresholds and then submitted to the catalepsy test, with stimulation being continued throughout testing. Current pulses were delivered by connecting rats to a pulse generator via cable using a tethered stimulation system. The procedure in sham-DBS controls (*n* = 7) was identical except that no current was administered. When treated with saline, all rats readily stepped down from the bar within few seconds (Fig. 1c, dotted line), with step-down latencies not differing between 30 Hz-DBS and sham-DBS control (*U* = 0.900; *P* = 0.408). Haloperidol treatment on the next day resulted in a marked increase in the step-down latency compared to saline administration in both the sham-DBS (*U* = 2.366; *P* = 0.018) and 30 Hz-DBS group (*U* = 2.666; *P* = 0.008) during a pretest without stimulation, indicating deep catalepsy. We then stimulated rats with 30 Hz-DBS of the IC and re-assessed the time spent in haloperidol-induced catalepsy (Fig. 1b). We found that haloperidol-induced catalepsy decreased by more than 50% following 30 Hz-DBS of the IC (*U* = 2.380; *P* = 0.017), resulting in a clear reduction compared to sham-DBS (*U* = 2.818; *P* = 0.005) where no change was evident (*U* = 1.000; *P* = 0.317; Fig. 1c), demonstrating a strong anti-cataleptic effect of our DBS protocol. Although intracollicular stimulation was unilateral, we observed no signs of lateralization in the anticataleptic effect.

### Low-frequency deep brain stimulation of the IC is not aversive

**Figure 2:**
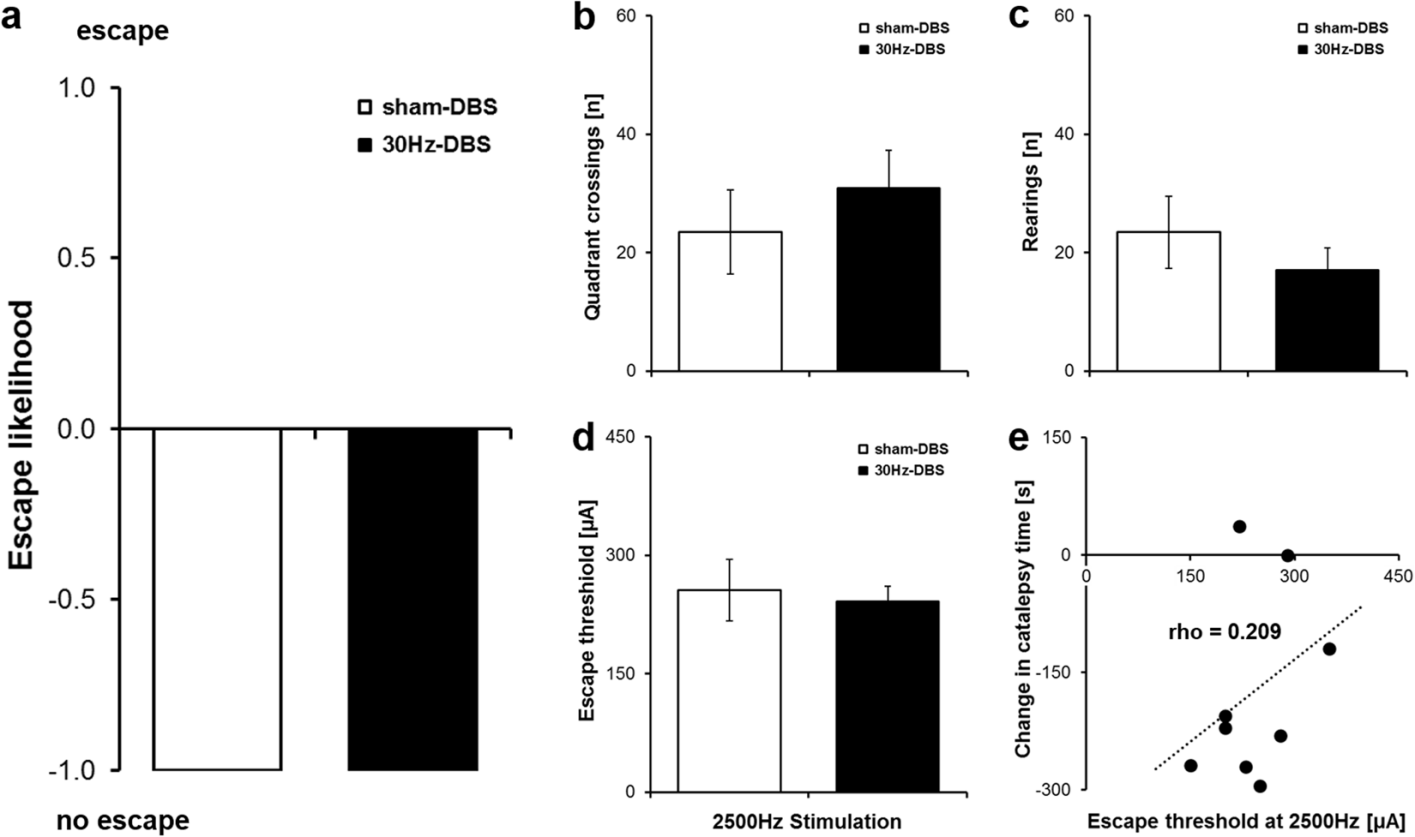
30 Hz deep brain stimulation of the IC does not induce aversive behaviour. **(a**) Escape likelihood as determined by the ratio of rats showing escape vs. no escape behaviour in the open field during 5 min sham-DBS (white) or 30 Hz-DBS (black), ranging from 1 (all rats showing escape) to -1 (no rat showing escape). Escape behaviour was completely absent in all rats of both groups. **(b)** Number of quadrant crossings during the open field saline session. Quadrant crossings did not differ between 5 min 30 Hz-DBS and sham-DBS. **(c)** Number of rearings during the open field saline session. Rearings were highly comparable between 5 min 30 Hz-DBS and sham-DBS. **(d)** Escape thresholds (minimum current intensity producing intense jumping or running) determined during 2500 Hz stimulation of the IC. Escape thresholds did not differ between groups. **(e)** Scatterplot depicting the relationship between individual escape thresholds at 2500 Hz (x-axis) and the change in the time spent in haloperidol-induced catalepsy from baseline following 30 Hz-DBS of the IC (y-axis). No correlation was found. Data are presented as mean ± s.e.m. *n* = 5–7 for sham-DBS, *n* = 9 for 30 Hz-DBS. *P < 0.05 vs. sham-DBS.

Importantly, the anti-cataleptic effect was not paralleled by the occurrence of escape behaviour. Thus, the escape-eliciting effect of stimulation at individual 2500 Hz escape thresholds was completely abolished in all rats when switching to 5 min low-frequency 30 Hz-DBS (Fig. 2a), indicating that our new protocol is not aversive. Indeed, open field exploratory activity, i.e. quadrant crossings (*U* = 0.953, *P* = 0.340) and rearings (*U* = 0.795, *P* = 0.427), was preserved in saline-treated rats during 30 Hz-DBS of the IC compared to sham-DBS (Fig. 2b,c). Moreover, escape thresholds at 2500 Hz stimulation were highly comparable between groups (*U* = 0.401, *P* = 0.688; Fig. 2d) and uncorrelated with the decrease in haloperidol-induced catalepsy following 30 Hz-DBS of the IC (rho = 0.209, *P* = 0.589; Fig. 2e), suggesting that the motor improvements in the 30 Hz-DBS group were not dependent on individual escape sensitivity. Together, these data indicate that sub-chronic 30 Hz-DBS of the IC strongly ameliorated haloperidol-induced catalepsy without any evidence of stimulation-induced aversive behaviour or acute motoric side effects.

**Figure 3:**
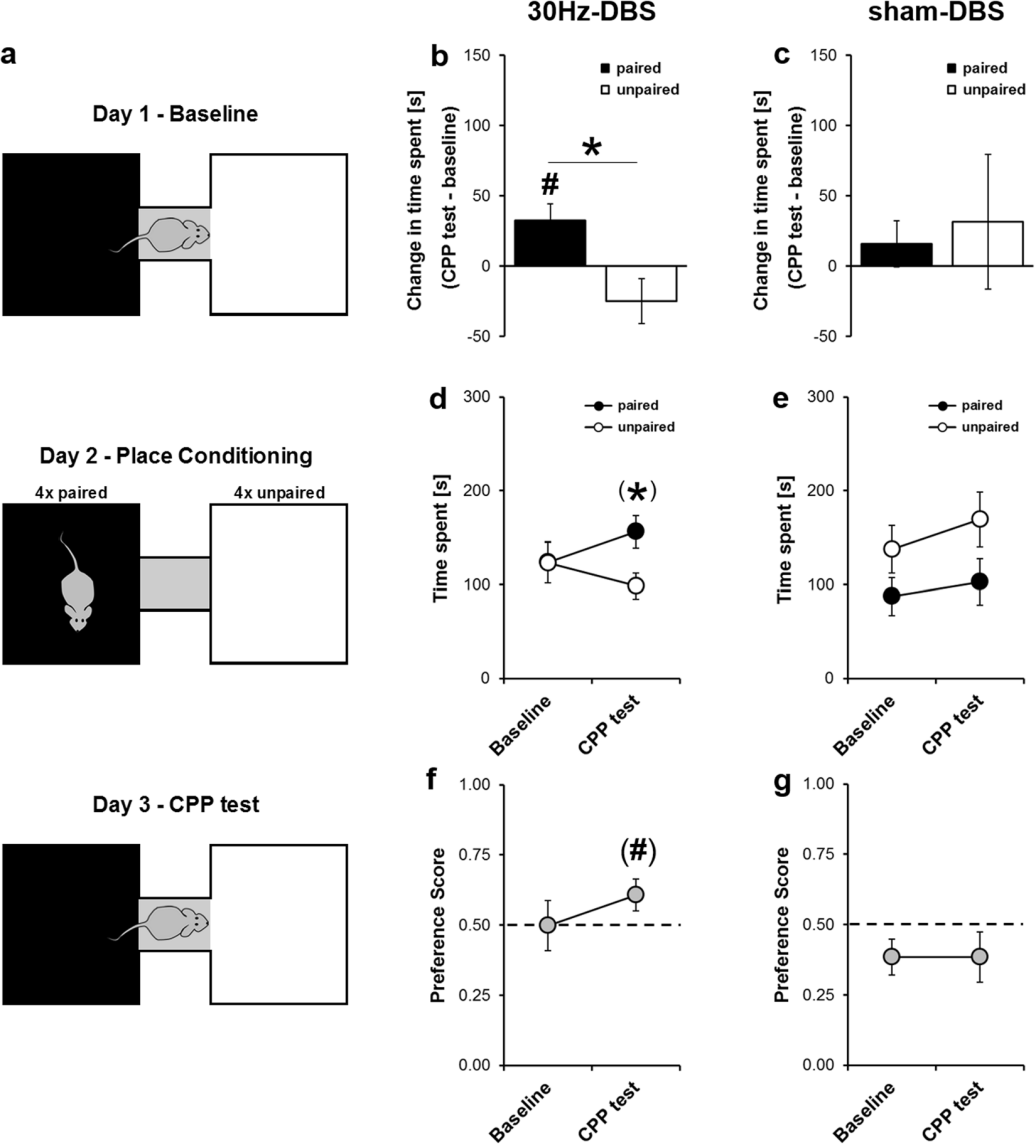
30 Hz deep brain stimulation of the IC promotes a mild form of conditioned place preference. **(a)** Illustration of conditioned place preference (CPP) protocol, consisting of a 5 min baseline session on day 1, 4 × paired (black) and unpaired (white) 5 min conditioning trials on day 2, and a 5 min CPP test on day 3. **(b,c)** Differences cores (CPP test – baseline) reflecting the change in time spent on the stimulation-paired and unpaired side from baseline to CPP test. **(b)** The time spent on the paired side significantly increased following place conditioning with 30 Hz-DBS of the IC, resulting a clear difference from the change in time spent on the unpaired side, i.e. where rats received sham-DBS and a non-significant decrease was seen. **(c)** No such effects were seen following sham-DBS. **(d,e)** Time spent on the stimulation-paired and unpaired side during baseline and CPP test. **(d)** 30 Hz-DBS of the IC promoted a mild preference for the stimulation-paired side during CPP test. **(e)** No such preference was seen following in rats receiving sham-DBS on both side chambers. **(f,g)** Preference scores [time paired/(time paired + unpaired)] during baseline and CPP test. **(f)** Preference scores slightly increased from baseline to CPP test in the 30 Hz-DBS group. **(g)** No such change was seen in sham-DBS controls. Data are presented as mean ± s.e.m. *n* = 5 for sham-DBS, *n* = 10 for 30 Hz-DBS. ^#^*P* < 0.05, ^(#)^*P* < 0.10 vs. baseline, *P < 0.05, ^(^*^)^*P* < 0.10 vs. unpaired.

We explored possible aversive side effects in more detail by testing the same animals in a conditioned place preference (CPP) paradigm. Place conditioning commenced after a 2-day washout period following haloperidol treatment and consisted of a 5 min baseline recording on day 1, followed by 8 5 min conditioning trials on day 2 using an unbiased design (*n* = 4 paired and unpaired trials, paired sides counterbalanced between rats), and a 5 min CPP test on day 3 (Fig. 3a). Specifically, rats were administered 30 Hz-DBS of the IC on paired trials, while receiving no stimulation, i.e. sham-DBS, on unpaired trials. Importantly, low-frequency 30 Hz-DBS of the IC (*n* = 10) produced no avoidance of the stimulation-paired side following conditioning. In fact, we rather obtained evidence for a mild form of place preference (time: *F*_(1,9)_ = 0.240, *P* = 0.634; side: *F*_(1,9)_ = 0.733, *P* = 0.414; time × side: *F*_(1,9)_ = 6.007, *P* = 0.037). Specifically, the time spent on the stimulation-paired side increased from baseline to CPP test in the 30 Hz-DBS group (*t*_9_ = −2.743, *P* = 0.023), being clearly different from the change in time spent on the unpaired side (*t*_9_ = 2.451, *P* = 0.037), where a slight, yet non-significant decrease was seen (*t*_9_ = 1.539, *P* = 0.158; Fig. 3b). Thus, 30 Hz-DBS conditioned rats showed a mild preference for the stimulation-paired side specifically during the CPP test (*t*_9_ = 1.887, *P* = 0.092) but not baseline (*t*_9_ = 0.009, *P* = 0.993; Fig. 3d). Consistently, there was a slight increase in preference scores for the stimulation-paired side from baseline to CPP test following place conditioning with 30 Hz-DBS of the IC (*t*_9_ = 1.976, *P* = 0.080; Fig. 3f). Moreover, compartment crossings and rearings increased from baseline to CPP test (Supplementary Fig. S1), possibly indicating enhanced anticipatory activity in 30 Hz-DBS conditioned rats. No such effects were seen in the sham-DBS group, i.e. where sham-DBS was administered on both side chambers (all *P*-values > 0.05, *n* = 5; Fig. 3c,e,g; Supplementary Fig. S1). Of note, sham-DBS controls displayed a bias in side preference, possibly caused by inefficient counterbalancing due to the small group size. However, this bias was constant, with preference scores staying nearly identical between baseline and CPP test (Fig. 3g). Importantly, no bias was seen in 30 Hz-DBS conditioned rats (Fig. 3f), indicating that indeed an unbiased design was employed when sample size was sufficiently high. Together, these data indicate that 30 Hz-DBS of the IC does not induce place avoidance and may rather promote a mild form of place preference.

**Figure 4:**
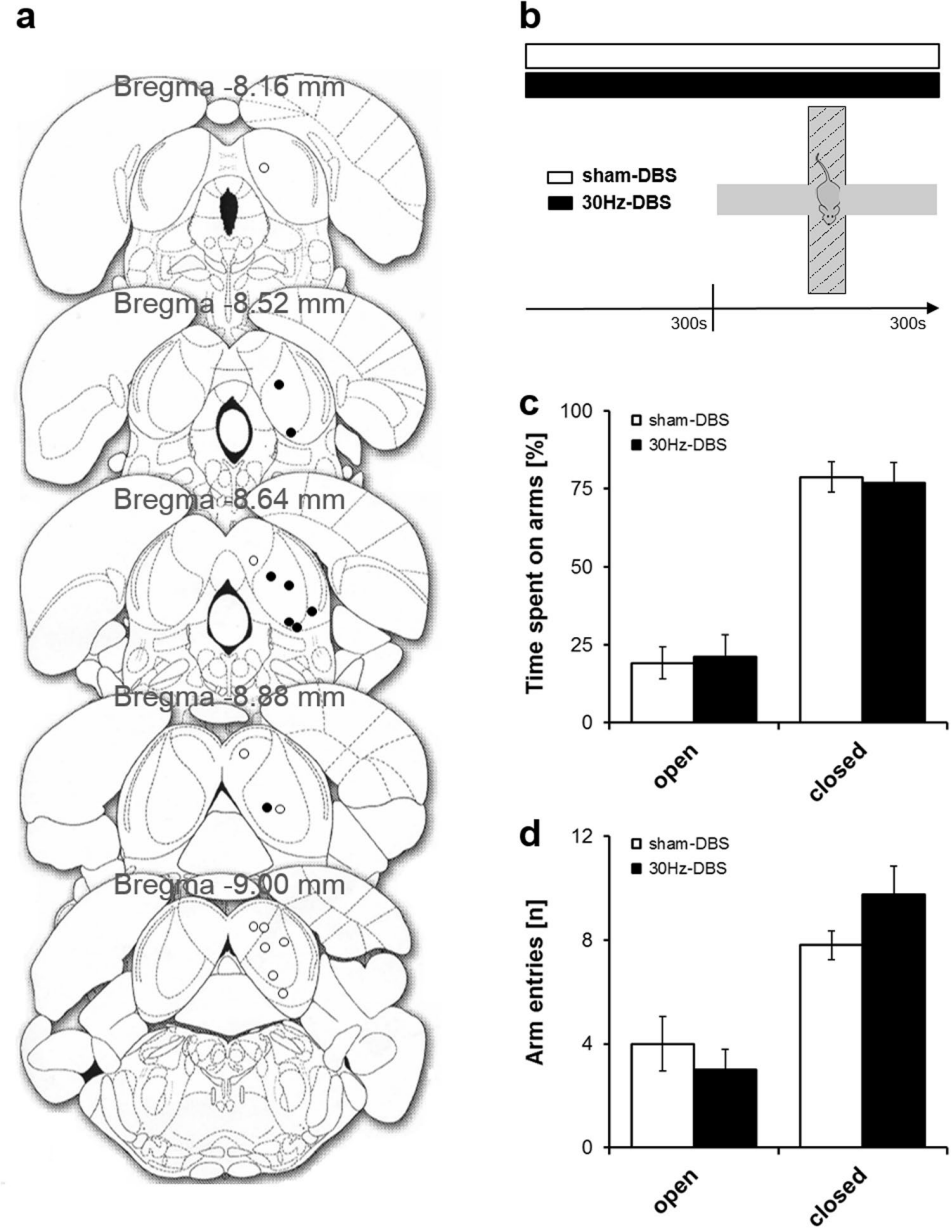
30 Hz deep brain stimulation of the IC induces no anxiety-related behaviour. (**a**) Electrode placements in the IC for 30 Hz-DBS (black dots) and sham-DBS (white dots) on coronal sections from the Paxinos and Watson^24^ atlas. **(b)** Illustration of the elevated plus maze (EPM) protocol. Rats were first stimulated sub-chronically for 5 min with 30 Hz-DBS of the IC and submitted to the EPM for a duration of 5 min, with stimulation being continued throughout testing. The procedure in sham-DBS controls was identical except that no current was administered. **(c)** Percent time spent on open and closed arms. (**d**) Open and closed arm entries. There were no significant differences between 30 Hz-DBS and sham-DBS control. Data are presented as mean ± s.e.m. *n* = 10 for sham-DBS, *n* = 8 for 30 Hz-DBS. *P < 0.05 vs. sham-DBS.

In an independent cohort of rats, we additionally tested the effects of our 30 Hz-DBS protocol on anxiety-related behaviour on the elevated plus maze (EPM)^20^. With the tethered system used so far, we reasoned that stimulation cables can get stuck while animals explore the maze, increasing the risk of cable disconnections and/or preventing full expression of the animal’s behavioural repertoire. We therefore switched to a newly developed telemetric system with similar performance as the tethered system and only minimal impact on the animal’s behaviour^21^. Again, 30 Hz-DBS was administered to the IC (Fig. 4a) at individual escape thresholds determined during 2500 Hz stimulation and carried out sub-chronically for 5 min before EPM, with 30 Hz stimulation being continued throughout testing (Fig. 4b). We observed no difference between 30 Hz-DBS (*n* = 8) and sham-DBS control (*n* = 10) in both the time spent on open (*t*_16_ = −0.248, *P* = 0.807) and closed arms (*t*_16_ = −0.232, *P* = 0.820) as well as in open (*t*_16_ = −0.728, *P* = 0.477) and closed arm entries (*t*_16_ = 1.704, *P* = 0.108; Fig. 4c,d), indicating that our 30 Hz-DBS protocol induced no anxiety-related behaviour. In line with this, there were no differences in additional parameters of anxiety-related behaviour, i.e. head dipping, scanning, risk assessment, and end arm exploration (all *P*-values > 0.05; data not shown). Thus, these results demonstrate that 30 Hz-DBS of the IC exerted no aversive or anxiogenic effects on the EPM, which is consistent with our open field and CPP data.

## Discussion

DBS has evolved as a promising treatment strategy for severe, medication-resistant cases of PD^1^. However, the underlying mechanisms remain poorly understood and conventional DBS protocols at basal ganglia sites can turn out as completely inefficient for some PD patients^1,4^, warranting the search for alternative targets. Here, we provide first evidence that the IC, a midbrain auditory relay structure, can serve as an alternative, non-conventional target for DBS.

We found that low-frequency 30 Hz-DBS targeted at the IC strongly ameliorates haloperidol-induced catalepsy. This finding demonstrates that the IC has a modulatory role in motor function and can be targeted with DBS to alleviate akinesia-like deficits in an acute pharmacological model with relevance to symptoms seen in PD^11^. Indeed, the IC has widespread indirect projections to structures involved in motor behaviour and movement coordination, including striatum, motor cortex, and cerebellum, all of which could be recruited by DBS to either restore or circumvent basal ganglia dopamine dysfunction^5,9,10,15^. Of note, we observed marked improvements despite deep catalepsy and although only unilateral stimulation was used. Clinical studies showed that bilateral DBS at basal ganglia structures is usually superior to unilateral DBS in the treatment of PD^22^. Thus, our protocol might be even more effective when using a bilateral stimulation approach.

Importantly, the motor improvements occurred without any evidence of stimulation-induced escape behaviour, indicating that our protocol is not aversive. In line with this, 30 Hz-DBS of the IC induced no place avoidance in the CPP test, produced no anxiety-related behaviour on the EPM, and did not trigger any other signs of aberrant motor reactions in the open field. Thus, we obtained no evidence of aversive or anxiogenic side effects across several independent measures. Notably, the IC is considered as part of the brain defence system and electrical stimulation of this structure thus far has only been associated with aversive behaviour^12–14^. In the present study, we now demonstrate that only by decreasing the stimulation frequency, electrical stimulation of the IC retained its modulating effects on motor behaviour while sparing out any additional aversive responses. Indeed, the improvement in haloperidol-induced catalepsy following 30 Hz-DBS of the IC was completely uncorrelated with individual differences in escape thresholds and thus aversive processing at the level of the IC. Our findings therefore possibly indicate a specific function of the IC in sensorimotor processing that can operate independently of the brain defence system. If any, we rather found some signs of positive side effects, as indicated by a mild form of conditioned place preference induced by 30 Hz-DBS of the IC. To the best of our knowledge, this is the first study indicating that electrical stimulation of the IC can be appetitive rather than aversive. This finding has therefore also important clinical implications given that PD is frequently associated with depression, with conventional DBS protocols targeted at basal ganglia sites often exacerbating such symptoms^4,23^.

## Conclusions

Together, we found that low-frequency 30 Hz-DBS of the IC strongly ameliorates haloperidol-induced catalepsy without any evidence of aversive side effects. These findings provide first evidence that the IC could serve as an alternative, non-conventional target for DBS in the treatment of PD.

## Materials and Methods

### Animals

Drug-naïve young adult male Wistar rats (Charles-River, Sulzfeld, Germany) were housed in single Macrolon Type III cages (L: 22 cm × W: 38 cm × H: 38 cm; with high acrylic cover) under controlled laboratory conditions (23 °C temperature, 40–60% humidity, and 12/12 day/night-cycle with lights on at 07:00). Food and water were available *ad libitum*. All protocols were in accordance with the current European guidelines and approved by the ethics committee of the local government (Regierungspräsidium Gießen; TVA G53–2016).

### Drugs

Haloperidol (0.5 mg/kg; Janssen Pharmaceutica, Beerse, Belgium) was diluted in physiological saline solution and administered intraperitoneally (i.p.) at a volume of 1 ml/kg.

### Electrode implantation

Stereotactic surgery was performed under isoflurane anaesthesia (Baxter Deutschland GmbH, Germany). A stimulation electrode (platinum electrode, iridium oxide coated, 250 μm outer diameter; Thomas RECORDING GmbH, Germany) was introduced vertically into the central nucleus of the IC using the following coordinates with lambda as reference from Paxinos and Watson^24^: anteroposterior = 1.2 mm; mediolateral = −1.5 mm; and dorsoventral = 4.5 mm. The electrode was fixed to the skull with acrylic resin and 3–4 stainless steel screws. Following surgery, 0.3 ml tramadol was administered subcutaneously (s.c.) to ensure analgesia. Rats were given a 7-day recovery period before start of behavioural experiments.

### Escape threshold determination and electrical stimulation

Individual escape thresholds were determined in an open field (40 × 40 × 40 cm) under red light (~28 lux) according to a previously established protocol^15^. For escape threshold determination, rats received high-frequency 2500 Hz stimulation (pulse width: 100 μs; pulse interval: 100 μs) to the IC in 1 min intervals. The current intensity was increased by 20–50 μA steps until rats showed escape behaviour, with stimulation being switched off immediately. The current intensity producing intense running or jumping was considered the escape threshold. Depending on experiment, electrical currents were either delivered by connecting stimulation electrodes to a pulse generator via cable using a tethered system (STG3008-FA, Multichannel Systems, Germany; Experiment 1) or to a wireless head stage targeted by a telemetric system (Thomas Wireless System, Thomas RECORDING GmbH, Germany; Experiment 2). Both systems have equal performance in electrical stimulation and electrophysiological recordings^21^, yet the telemetric system was chosen for Experiment 2 since no cables are required that could possibly get disconnected or get stuck in the closed arms of the elevated plus maze.

## Experiment 1: Effects of 30 Hz deep brain stimulation of the IC in haloperidol-induced catalepsy and place conditioning

### General procedur**e**

Rats were assigned to 30 Hz-DBS (*n* = 12) or sham-DBS (*n* = 7). Stimulation intensity was determined individually by assessing each rat’s threshold for escape behaviour during high-frequency 2500 Hz stimulation of the IC. Animals were then tested in the catalepsy test under either 30 Hz- or sham-DBS at the IC on two consecutive days following saline and haloperidol administration. After a 2-day washout period, rats were subjected to a conditioned place preference (CPP) paradigm to assess possible aversive side effects of 30 Hz-DBS. Before CPP test, escape threshold intensities were re-determined to account for possible effects of electrode displacement. All rats were handled for 5 min on three consecutive days before start of behavioural experiments.

### Catalepsy test

Catalepsy was assessed in an open field (40 × 40 × 40 cm) under red light (~28 lux) on two consecutive days following saline and haloperidol administration, respectively, according to a modified protocol^15^. Sixty min after injection, the animal’s forepaws were positioned on a horizontal bar elevated 8 cm above the floor and the step-down latency was recorded on three successive trials, using a stop criterion of 300 s. For haloperidol-treated rats, DBS procedure started after the animal remained for at least 30 s in catalepsy, i.e. with its forepaws on the horizontal bar, during three consecutive trials. 30 Hz-DBS was delivered sub-chronically at individual escape thresholds for 5 min before testing and was continued throughout the catalepsy test. During the first 5 min of DBS procedure the number of crossings and rearings was assessed. The procedure in sham-DBS control animals was identical except that no current was applied. Behaviour was recorded with a video camera (EverFocus, model: EQ. 150) centrally placed above the open field.

### Conditioned place preference

Place conditioning was conducted in a three-chamber box (side chambers: 23 × 35 × 35 cm; central corridor: 12 × 10 × 35 cm) under red light (~30 lux) on three consecutive days. On day 1 (baseline), rats had free access to the chambers and baseline preference was assessed during a 5 min recording. On day 2 (conditioning), place conditioning was carried out on eight successive 5 min trials. Rats of the 30 Hz-DBS group were confined to one of the side chambers. On alternating trials, rats received sub-chronic 30 Hz-DBS (4×) in the stimulation-paired side and no stimulation, i.e. sham-DBS, (4×) in the unpaired side for a duration of 5 min each. An unbiased place conditioning design was used, with stimulation-paired sides being counterbalanced between rats^25–27^. Floor texture and visual patterning differed between the two side chambers to ensure discrimination. Paired and unpaired trials were separated by a 5 min inter-trial interval to account for possible carry-over effects of 30 Hz-DBS. The procedure in sham-DBS controls was identical except that no stimulation was applied, i.e. rats received sham-DBS in both side chambers. On day 3 (test), rats had again free access to the chambers and preferences were re-assessed during a 5 min recording. Time spent in the side chambers, compartment crossings, and rearings were recorded with a video camera (Security Center TV70150; CN: AR08175; 12VDC/2.1 W) mounted centrally above the three-chamber box.

## Experiment 2: Effects of 30 Hz deep brain stimulation of the IC on anxiety-related behaviour

### General procedure

Rats were assigned to 30 Hz-DBS (*n* = 8) or sham-DBS (*n* = 10). As for Experiment 1, the stimulation intensity was determined individually by assessing each rat’s threshold for escape behaviour during high-frequency 2500 Hz stimulation of the IC. Animals were then submitted to the elevated plus maze (EPM) under either 30 Hz- or sham-DBS at the IC to test for possible effects on anxiety-related behaviour.

### Elevated plus maze

The EPM consisted of two open (50 × 10 cm) and two closed arms (50 × 10 cm, with 40 cm high walls) extending from a central platform elevated 50 cm above the floor. Rats were connected to the telemetric system and first stimulated sub-chronically for 5 min with 30 Hz-DBS of IC at individual escape thresholds in a clean home cage. Thereafter, animals were submitted to the EPM, with stimulation being continued throughout testing. Specifically, each rat was placed onto the central platform of the EPM with its head facing one of the open arms and then allowed to freely explore the open and closed arms for a duration of 5 min according to previously used protocol^21^. The procedure in sham-DBS controls was identical except that no current was administered. Time spent on the open and closed arms as well as open and closed arm entries were recorded with a video camera (Panasonic WVBP330/GE, Hamburg, Germany) mounted centrally above the arena. Additionally, the following anxiety-related behaviours were assessed: (I) head dipping (dipping the head below the maze floor), (II) scanning (horizontal head movements in any direction, including sniffing the maze floor and walls), (III) risk assessment (stretched-attend postures into the open arms, with hind paws staying in the closed arm), (IV) and end arm exploration (reaching the end of the open arms)^20,28^.

### Histology

At the end of all behavioural experiments, the animals were deeply anesthetized with xylazine/ketamine (300 mg/kg and 200 mg/kg, respectively; i.p.) and perfused through the left ventricle. The blood was washed out with physiological saline followed by 200 ml 4% (w/v) paraformaldehyde in 0.1 M sodium phosphate buffer, pH 7.3. The midbrains were quickly removed and immersed for 4 h in fresh fixative at 4 °C. After fixation, the brains were frozen and 50 μm serial brain sections were cut using a microtome. The sections were stained with cresylviolett in order to locate the positions of the electrode tips, according to the atlas by Paxinos and Watson^24^. Data from rats with tips located outside the IC were not included in the statistical analysis.

### Statistical analysis

Data from the catalepsy test were analysed with Wilcoxon signed-rank and Mann-Whitney U tests, with three rats of the 30 Hz-DBS group being excluded from analysis due to data loss. For CPP, an unbiased design was used, which allows testing for conditioned side preferences using within-subject comparisons^25–27^. Therefore, CPP data were analysed separately for sham- and 30 Hz-DBS groups using repeated-measures analyses of variance (ANOVAs), with time (baseline vs. test) and side (paired vs. unpaired) serving as within-subject factors. ANOVAs were followed by paired *t*-tests when appropriate. Additionally, a preference score for the stimulation-paired side was calculated using the following equation: preference score = [time paired/(time paired + unpaired)]. The preference score was compared between baseline and CPP test. Behaviour was analysed when a rat explored both side chambers during baseline, with two sham-DBS animals and one 30 Hz-DBS animal not fulfilling the criterion and thus being excluded from analysis. One rat of the 30 Hz-DBS group had to be excluded from analysis due to data loss. EPM data were analysed by comparing 30 Hz-DBS and sham-DBS group using unpaired *t*-tests.

### Data availability

The datasets generated during and/or analysed during the current study are available from the corresponding author on reasonable request.

## Electronic supplementary material

Supplementary Figure S1
