# Supplementary material for: Haloperidol–induced catalepsy is ameliorated by deep brain stimulation of the inferior colliculus
Source: Sci Rep. 2018 Feb 2;8:2216. doi: 10.1038/s41598-018-19990-y (PMC5797241; doi:10.1038/s41598-018-19990-y)
Supplement: Supplementary file 1 — Supplementary Figure S1 [file 41598_2018_19990_MOESM1_ESM.doc]

**Supplementary Figure S1**

**Haloperidol–induced catalepsy is ameliorated by deep brain stimulation of the inferior colliculus**

K.-Alexander Engelhardt^1^, Philine Marchetta^1^, Rainer K. W. Schwarting^1,2^ & Liana Melo-Thomas^1,2,3,*^

^1^ Behavioral Neuroscience, Experimental and Biological Psychology,

Philipps-University of Marburg, Gutenbergstr. 18, D-35032 Marburg, Germany

^2^Marburg Center for Mind, Brain, and Behavior (MCMBB), Hans-Meerwein-Straße 6, 35032 Marburg, Germany

^3^Behavioral Neurosciences Institute (INeC), Av. do Café, 2450, Monte Alegre, Ribeirão Preto, 14050-220, São Paulo, Brazil.

*Correspondence should be addressed to:

Liana Melo-Thomas

Behavioral Neuroscience

Experimental and Biological Psychology

Philipps-University of Marburg

Gutenbergstr. 18, 35032 Marburg, Germany

Fax: +6421 28 23610, Tel: +6421 28 23694

e-mail: liana.melothomas@staff.uni-marburg.de


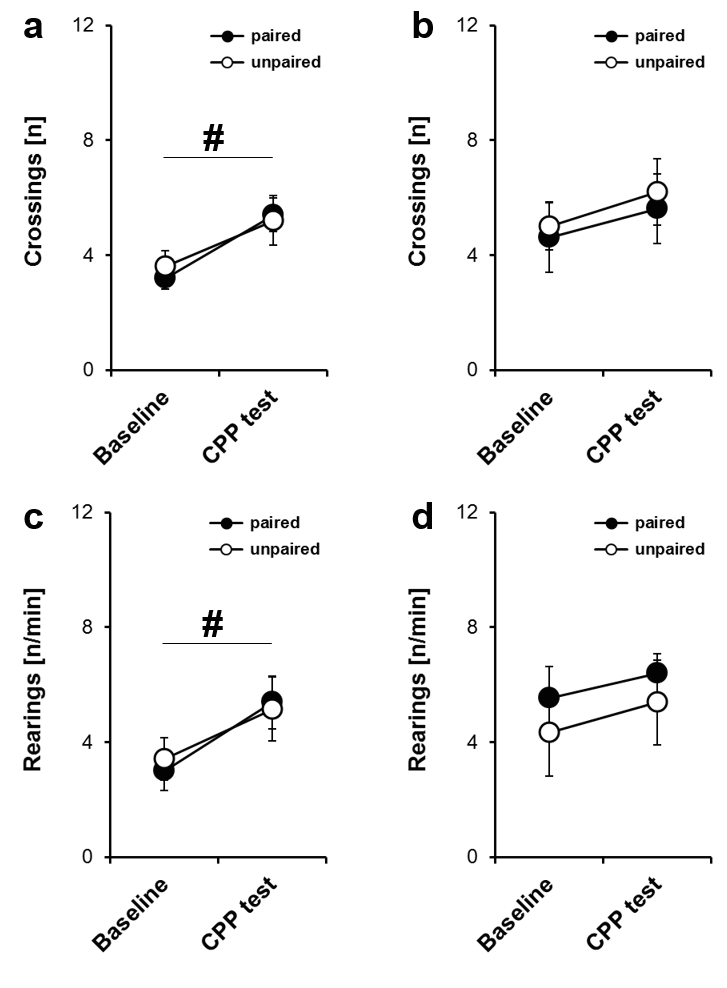


**Supplementary Figure S1: Mildly enhanced exploratory activity following place conditioning with 30Hz deep brain stimulation of the IC. (a-b)** Compartment crossings and **(c-d)** rearings per time spent in the stimulation-paired (black) and unpaired (white) side in 30Hz- and sham-DBS conditioned rats. **(a)** Compartment crossings (*F*_(1,9)_=8.042, *P*=0.020) and **(c)** rearings (*F*_(1,9)_=9.394, *P*=0.013) showed a slight increase from baseline to CPP test following place conditioning with 30Hz-DBS of the IC, indicating mildly enhanced exploratory activity in 30Hz-DBS conditioned rats. **(b, d)** No such changes were seen in rats conditioned with sham-DBS (all *P*-values >0.05). Data are presented as mean ± s.e.m. *n*=5 for sham-DBS, *n*=10 for 30Hz-DBS. #*P*<0.05 vs. baseline.
